# Comparison of three methods of EPR retrospective dosimetry in watch glass

**DOI:** 10.3389/fpubh.2022.1063769

**Published:** 2022-11-17

**Authors:** Agnieszka Marciniak, Małgorzata Juniewicz, Bartłomiej Ciesielski, Anita Prawdzik-Dampc, Jakub Karczewski

**Affiliations:** ^1^Department of Physics and Biophysics, Medical University of Gdańsk, Gdańsk, Poland; ^2^Department of Oncology and Radiotherapy, Medical University of Gdańsk, Gdańsk, Poland; ^3^Institute of Nanotechnology and Materials Engineering, Faculty of Applied Physics and Mathematics, Gdańsk University of Technology, Gdańsk, Poland

**Keywords:** EPR, dosimetry, glass, radiation, dose, annealing

## Abstract

In this article we present results of our follow-up studies of samples of watch glass obtained and examined within a framework of international intercomparison dosimetry project RENEB ILC 2021. We present three methods of dose reconstruction based on EPR measurements of these samples: calibration method (CM), added dose method (ADM) and added dose&heating method (ADHM). The study showed that the three methods of dose reconstruction gave reliable and similar results in 0.5–6.0 Gy dose range, with accuracy better than 10%. The ADHM is the only one applicable in a real scenario, when sample-specific background spectrum is not available; therefore, a positive verification of this method is important for future use of EPR dosimetry in glass in potential radiation accidents.

## Introduction

The increasing use of ionizing radiation in industry, medicine and other areas of everyday life causes the need to control exposure of people to this factor. Despite of the growing awareness of people and use of various safety measures, radiation accidents may occur, where people may be irradiated with dangerously large doses of ionizing radiation. This raises the need for reliable methods of post-accident dosimetry useful in assessment of scale of the accident and in planning medical assistance to exposed people. One of such retrospective methods is based on electron paramagnetic resonance (EPR), which involves the detection and quantification of EPR signals from stable free radicals generated by ionizing radiation. So far, various materials have been studied, both biological, like tooth enamel, bones or nails (1–5), as well as those present in humans' environment (1, 6–14). Materials that come into close contact with humans, such as the screen of a mobile phone or the glass of a watch, are particularly attractive. They have many advantages for potential retrospective dosimetry, such as widespread availability, resistance to water, chemical inertness (10, 15, 16) and non-invasive sampling. However, the reliability of EPR dosimetry in glass requires consideration of several major confounding factors. One of them is the necessity of individual approach to each sample due to the differences in spectral characteristics of the native background (BG) signal and the radiation-induced signal (RIS) in different types of glasses. The more that their types are usually difficult to identify by simple methods. This is a significant obstacle in application of this method in a real scenario, when one can examine only the very sample, which had been irradiated in the accident and another, unirradiated samples of the same type of glass are not available. Consequently, the lack of information about the sample's background signal, which is crucial for accuracy of dosimetry, can prevent application of this method. The tests of retrospective EPR dosimetry in glasses presented so far in the literature (6) were carried out in laboratory conditions (i.e., using the CM), when the background signal of the tested glass was available and dose calibration was done by irradiation of unexposed glass of the same type.

Annealing of irradiated glass samples at high temperatures (above 200 °C) in order to recover their BG signal by elimination of the dosimetric component (RIS) have already been proposed by various researchers in: watch glass (17), window glass (16, 18, 19), bioactive glass (Bio-G) (20), fused quartz (21), glass test tubes (22), mineral glass from mobile phone (8) and glass from phones' LCDs screens (10). However, applicability of this method, which can be successfully applied only, if the annealing bleaches out the RIS and does not affect the shape of BG signal, has not been yet verified experimentally.

This article presents results of verification of the procedure of dose reconstruction using three methods for watch glasses irradiated with an unknown dose of ionizing radiation: (1) calibration method (CM), (2) added dose method (ADM) and (3) added dose&heating method (ADHM).

In a real scenario of a radiation accident, it is the most probable that retrospective dosimetry would have to be based only on the samples irradiated in the accident, without a possibility to use unirradiated glass samples of the same type for determination of the BG signal and for calibration of the RIS. Therefore, in a real scenario the ADHM can be the only applicable method. In this article we compare results of retrospective dosimetry obtained with the three mentioned above methods.

## Materials and methods

### Samples

The examined watch glass samples were delivered by organizers of international inter-comparison project RENEB ILC 2021 (23) in which we participated.

The elemental composition of the watch glass, as determined by Energy Dispersive Spectroscopy (EDS) at the Institute of Nanotechnology and Materials Engineering of the Gdańsk University of Technology, was: 27.5% Si, 11.0% Na, 2.5% Mg, 1.0% Al, 1.0% K, 3.0% Ca and 54.0% O. The measurement uncertainty was 0.5% for all elements except oxygen, for which the uncertainty was 3.0%.

Before EPR measurements the glasses were cut into small pieces, crushed in agate mortar and sieved to the final grain size in the interval of 0.5–2 mm. It was reported, that such crushing did not generate any EPR signal in glass (1, 10, 15).

### EPR measurements and spectrometer settings

The EPR measurements were carried out at room temperature with a Bruker EMX-6/1 spectrometer in X-band with a cylindrical cavity of type 4119HS W1/0430 using the following acquisition parameters: 350.5 mT central magnetic field; 9.88 GHz microwave frequency; 32 mW microwave power; 100 kHz modulation frequency; 0.5 mT modulation amplitude; 163.84 ms time constant and 81.92 sweep time, 5 averaged scans per one spectrum. The 150–180 mg samples were positioned in the central region of the EPR cavity in a quartz EPR tube of 4 mm inner diameter. Each sample was measured at three orientations of the sample tube in the cavity and the spectra were averaged. Intracavity standard sample (Mn^2+^ in MgO) was measured simultaneously with all samples and can be seen as two sharp lines at the spectra wings in the presented signals (**Figures 3**, **5**, **6A**).

### Data analysis

Quantitative analysis of the spectra (alignment and normalization of their amplitudes to the standard's lines and sample mass, subtractions of the empty tube spectrum, averaging, numerical decompositions of the spectra) was carried out using Microsoft Office Excel 2016. Numerical fitting/decomposition of the experimental spectra into the BG and RIS components, as described in Marciniak et al. (12), was performed using the Reglinp procedure in Excel.

### Irradiations

All samples were irradiated at room temperature with single doses in a Maxishot SPE X-ray cabin (Yxlon, Hamburg, Germany) using 3 mm beryllium and 3 mm aluminum filters, an accelerating potential of 240 kV with half value layer (HVL) 0.630 ± 0.025 mm of copper^1^. The examined samples were of two types: blinded (with the doses revealed to participants only after reporting of the results), which were exposed to X-rays to doses 0 Gy, 1.2 Gy, and 3.5 Gy, and calibration samples irradiated with known doses of 0.0, 0.5, 1.0, 2.0, 3.0, and 6.0 Gy – in terms of kerma in air.

### Annealing of the samples

Annealing of unirradiated and irradiated samples was performed in a drying oven VWR VENTI-Line with Forced Convection (VL 53, VL 115) at temperature of 200 °C and in a furnace at 250 °C.

### Calibration method

The spectra decomposition was performed using the model BG signal measured in an unirradiated (0.0 Gy) sample of the same glass type and the model RIS, which was determined as difference between spectra of the 6.0 Gy and 0.0 Gy calibration samples. Magnitudes of the RIS components determined in the blinded samples were implemented into the calibration lines (represented by liner regression of data points in **Figure 4**) to reconstruct the unknown doses. The rate of decay of the RIS in time was determined by repetition of measurements of the irradiated samples in time (inset in **Figure 4**). This decay rate was applied to account for different periods between irradiation and EPR measurements of the blinded and calibration samples, as shown in the Results.

### Added dose method

Added dose method (ADM) is applicable when the BG signal is known and the very sample with the unknown dose is used to perform the RIS vs. dose calibration by its additional irradiation with a known dose, instead of the calibration based on separate, dedicated calibration samples.

The radiation sensitivity of the samples was individually calibrated by their re-irradiation with 6 MVp photons from Clinac 2300 (Department of Oncology and Radiotherapy, Medical University of Gdańsk, Poland). The added calibration dose (*D*_*cal*_) was (6.0 ± 0.1) Gy in terms of absorbed dose to water, which is equivalent to (5.17 ± 0.10) Gy in glass [this water-to-glass dose conversion was based on mass absorption coefficients and stopping powers from NIST website (https://www.nist.gov)].

In the spectra decomposition the model RIS was represented by the differential spectrum, i.e., the difference between the samples' EPR spectra measured after and before re-irradiation of the samples with the added dose *D*_*cal*_; the model BG was represented by the spectrum measured in the unirradiated (0.0 Gy) sample.

**Figure 1:**
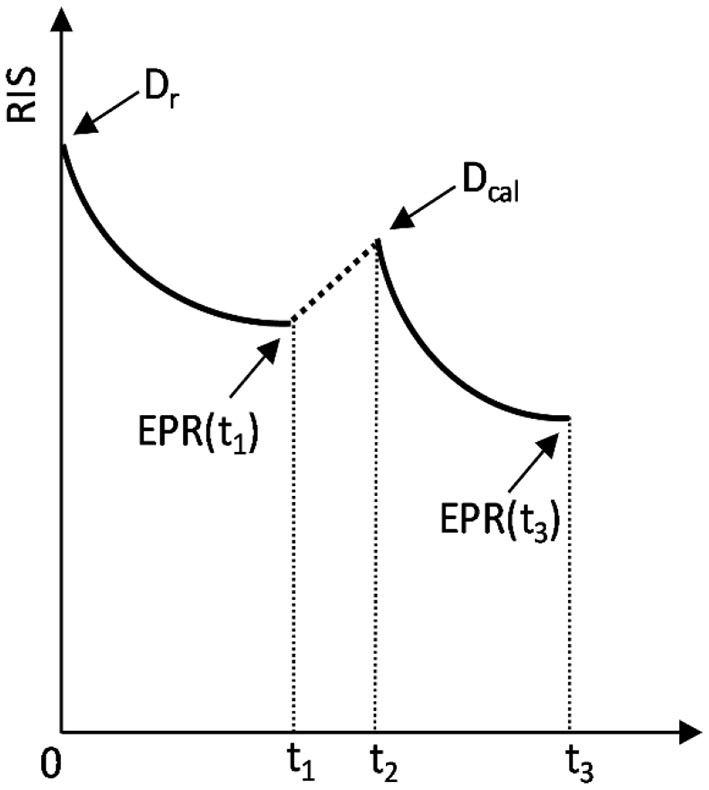
Schematic representation of the added dose method. The sample was irradiated at *t* = 0 with the unknown dose *D*_*r*_, measured at *t* = *t*_1_, re-irradiated at *t*_2_ with the added calibration dose *D*_*cal*_ and measured again at *t* = *t*_3_. The arrows marked as *EPR*(*t*_1_) and *EPR*(*t*_3_) indicate the two moments of EPR measurements: at *t*_1_ and *t*_3_, which yield two measured RISes: *RIS*(*D*_*r*_) and *RIS*(*D*_*r*_ + *D*_*cal*_), respectively.

The procedure of ADM is graphically presented in Figure 1.

The unknown dose *K*_*air*_ in terms of kerma in air can be reconstructed as follows:


(1) $$\begin{array}{l} RIS(D_{r})=k(t_{1})\;\cdot \;c\;\cdot \;D_{r} \end{array}$$

(2) $$\begin{array}{l} RIS\left(D_{r}+D_{cal}\right)=k\left(t_{3}\right)\cdot c\cdot D_{r}+k\left(t_{3}-t_{2}\right)\cdot c\cdot D_{cal} \end{array}$$

where:

*D*_*r*_ – the unknown dose in glass;

*c* – proportionality constant dependent on settings of EPR spectrometer and the spectra decomposition procedure – those conditions were the same for all measurements.

*RIS*(*D*_*r*_) – the value of the dosimetric signal generated by the unknown dose *D*_*r*_, measured before re-irradiation of the samples;

*k*(*t*)− a function representing decay of the RIS in time *t*; a single exponential decay was assumed, i.e., by the function $k\left(t\right)=a_{0}+a_{1}\cdot e^{-\frac{t}{a_{2}}}$, with *a*_0_ = 0.10607, *a*_1_ = 0.09368 and *a*_2_ = 20.48925 (inset in **Figure 4**) for time *t* given in days. The numerical values for *a*_0_, *a*_1_ and *a*_2_ were obtained from dependence of the slope of the calibration lines on time. Taking into account direct proportionality between the RIS and dose (as shown in **Figure 4**), relative changes in the RIS are the same, as relative changes in the slope. However, the changes in the slope (which is a resultant of several data points for various samples with different doses) gives a better statistics in the calculated rate of decay *k(t*), than calculations of the *k(t)* based on monitoring decay of RIS in just one dose or sample.

*D*_*cal*_ - the known added dose (*D*_*cal*_ = 5.17 *Gy* in glass);

*RIS*(*D*_*r*_ + *D_cal_*) – the value of the dosimetric signal measured after re-irradiation of the glass samples with the added calibration dose *D*_*cal*_.

The solution of those two equations yields:


(3) $$\begin{array}{l} D_{r}=D_{cal}\;\cdot \;\frac{RIS\left(D_{r}\right)\;\cdot \;k\left(t_{3}-t_{2}\right)}{RIS(D_{r}+D_{cal})\;\cdot \;k\left(t_{1}\right)-RIS(D_{r})\;\cdot \;k\left(t_{3}\right)} \end{array}$$

Under electronic equilibrium conditions, the dose *D*_*r*_ in glass can be expressed in terms of kerma in air *K*_*air*_:


(4) $$\begin{array}{l} D_{r}={\left(K\begin{array}{c} glass\\ air \end{array}\right)}_{240kV}\cdot K_{air} \end{array}$$

where the proportionality factor ${\left(K\begin{array}{c} glass\\ air \end{array}\right)}_{240kV}=2.43$ represents ratio of mass absorption coefficients of glass and air, calculated using data from NIST Standard Reference Database 124 and 126 (https://www.nist.gov) for air and the elements of glass at 66 keV (i.e., the photon energy with HVL= 0.63 mm Cu). Finally one gets:


(5) $$\begin{array}{l} K_{air}=\frac{D_{cal}}{{\left(K\begin{array}{c} glass\\ air \end{array}\right)}_{240kV}}\\ \;\cdot \;\frac{RIS(D_{r})\;\cdot \;k(t_{3}-t_{2})}{RIS\left(D_{r}+D_{cal}\right)\;\cdot \;k(t_{1})-RIS(D_{r})\;\cdot \;k(t_{3})} \end{array}$$

### Added dose&heating method

In this method (ADHM) the dose was reconstructed by samples' re-irradiation, as described above for the ADM, with the difference that the BG spectrum in the decomposition procedure was approximated by bleaching out the RIS in the irradiated samples by their annealing at 200 °C or at 250 °C for 4–60 min.

**Figure 2:**
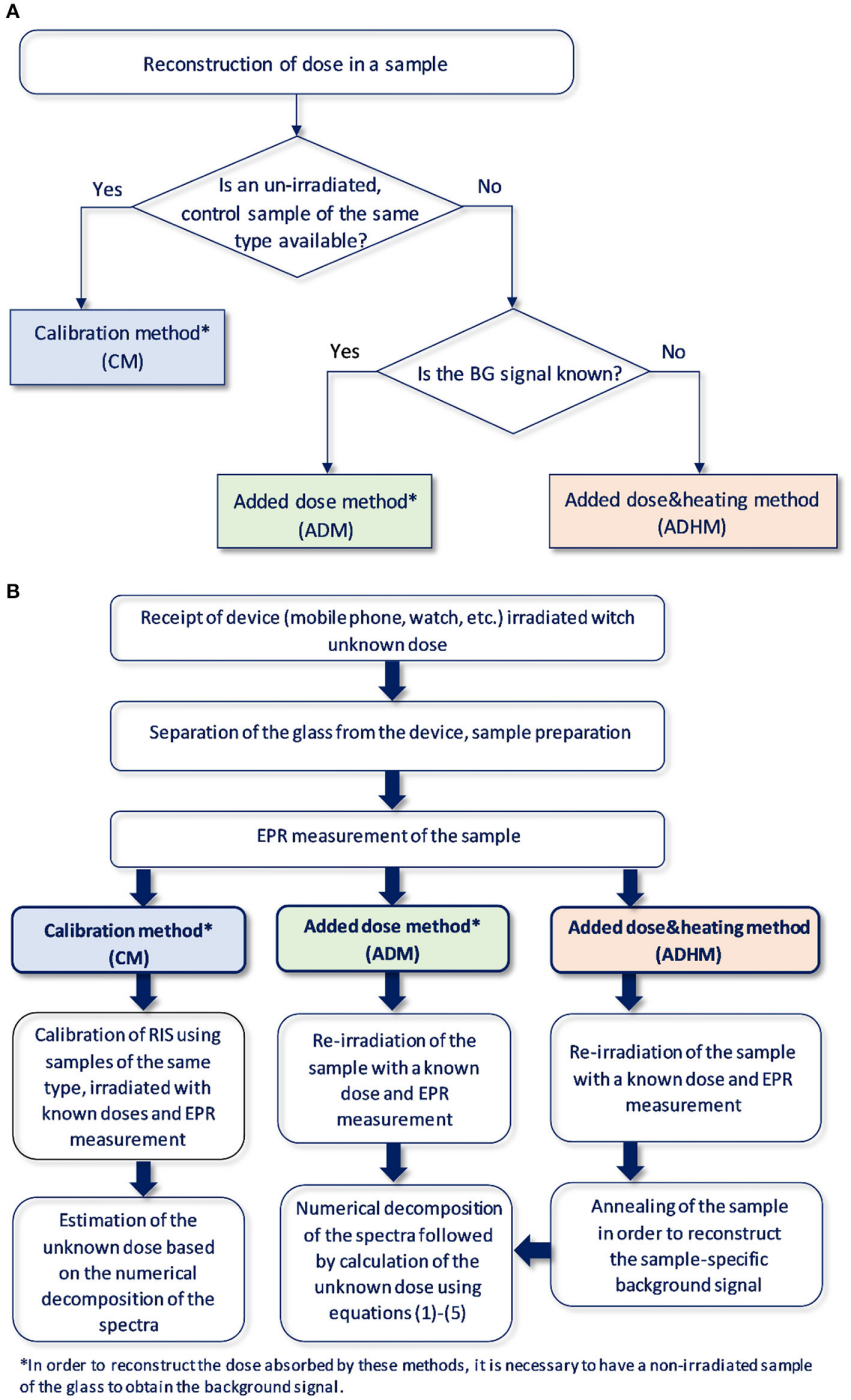
**(A)** Logical diagram for selection of the method of reconstructing the absorbed dose in the glass depending on the available information and the tested material. **(B)** Schematic illustration of the dose reconstruction procedure.

A summary of the above outlined dose reconstruction procedures is shown schematically in the Figure 2.

## Results

**Figure 3:**
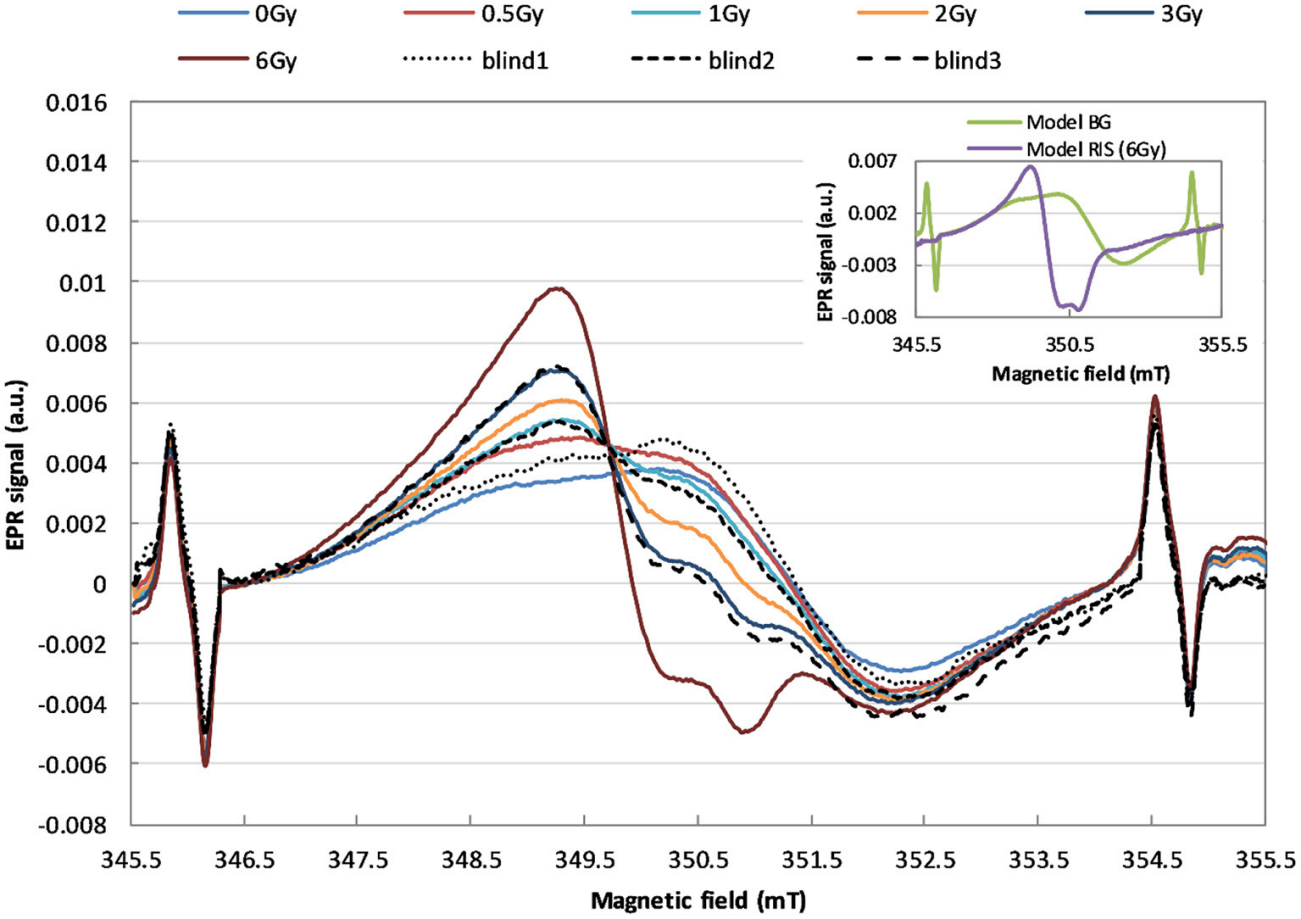
EPR spectra demonstrating the effect of irradiation of the watch glass with 240 keV X-rays with doses in the range of 0–6 Gy (in terms of kerma in air). The solid lines refer to the calibration samples, the dashed and dotted lines refer to the blind samples.

EPR spectra of the six calibration samples (0.0, 0.5, 1.0, 2.0, 3.0, and 6.0 Gy) and three blind samples (0.0, 1.2, and 3.5 Gy) are presented in Figure 3.

The two model spectral components, BG and RIS, which overlap with various relative contributions (depending on the dose) in those spectra, are shown in the inset in Figure 3.

The doses in the blind samples #1, #2, and #3 were determined with two methods based on knowledge of the BG signal, which was measured in an unirradiated glass sample: (1) the calibration method (CM) based on calibration of the RIS using the six calibration samples irradiated by organizers of the RENEB ILC with 240 kV X-rays (the same radiation as used for the blind samples), (2) the method of added dose (ADM) using re-irradiation of the samples with 6 Gy (in terms of dose in water) by 6 MV X-rays from the Clinac. In the added dose&heating method (ADHM), the BG spectrum used in numerical decomposition was approximated by EPR spectra from the irradiated samples, in which the RIS was bleached out by annealing at 200 or 250 °C.

### Calibration method

**Figure 4:**
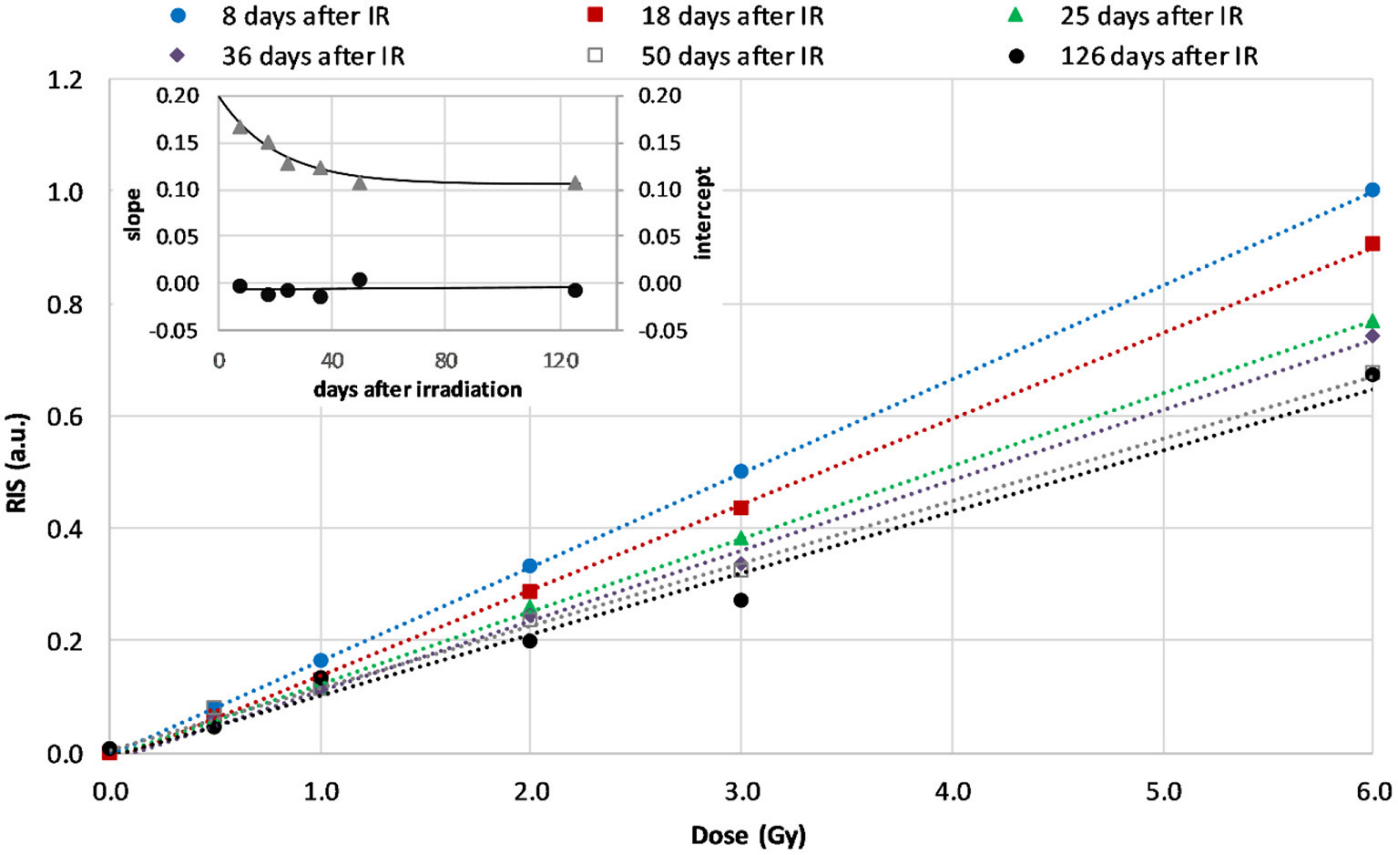
Calibration lines for WG measured at 8^th^, 18^th^, 25^th^, 36^th^, 50^th^, and 126^th^ day after irradiation (dashed lines are linear regressions of the data). The inset shows time dependence of the slopes (the rate of decay of the RIS, triangles) and intercepts (circles) of the regression lines in the first 126 days after irradiation.

Figure 4 shows the dose dependence of the RIS for the WG calibration samples measured 8, 18, 25, 36, 50, and 126 days after irradiation. The decay of RIS in time caused changes in the course of the calibration lines, mainly a drop in their slope while intercept of the regression lines shown in Figure 4 did not change (as shown in the inset). The dashed lines represent linear regression of the data. The uncertainty of the plotted data was less than 1%, therefore the error bars are not shown in Figure 4.

The dependence of the course of those lines (their slopes and intercepts) on time after irradiation was applied to reconstruct the doses in samples measured at days different than those reflected in Figure 4. The blind doses reported in the RENEB ILC were determined from measurements performed on 7^th^ and 11^th^ day and their averaged values are given in the third column of Table 1.

**Table 1:** Comparison of the reconstructed doses using calibration method (CM), added dose method (ADM) and added dose&heating method (ADHM).

**Samples**	**Real** ***K*_*air*_ [Gy]**	**Dose estimate** **by CM [Gy]**	**Dose estimate** **by ADM [Gy]**	**Dose estimate** **by ADHM [Gy]**
Blind 1	0	−0.05	0.63	
Blind 2	1.2	1.03	1.39	
Blind 3	3.5	3.16	3.70	3.19
Cal dose 0.5	0.5		0.47	
Cal dose 1.0	1.0		1.07	
Cal dose 3.0	3.0		3.12	

The gray shadow indicates empty fields in the table (with no data).

### Added dose method

From the values of the dosimetric signals [*RIS*(*D*_*r*_) and *RIS*(*D*_*r*_ + *D*_*cal*_)] determined in EPR spectra measured before and after re-irradiation with the known dose (*D*_*cal*_) the unknown doses were reconstructed using equations (1)–(5).

The fourth column of Table 1 presents doses reconstructed by the ADM following re-irradiation of the samples with *D*_*cal*_ = 5.17 *Gy* after 41 days (for the 0.5 Gy, 1 Gy and 3 Gy calibration samples) and 407 days (for the three blind samples) after the first irradiation of those samples by the 240 kV X-rays (during the RENEB ILC). Those re-irradiations were followed by EPR measurements on the next day – i.e. in the equation (5) the values of *t* were *t*_1_ = 8, *t*_2_ = 41, and *t*_3_ = 42 days for the 0.5 Gy, 1 Gy, and 3 Gy calibration samples and *t*_1_ = 407, *t*_2_ = 407, and *t*_3_ = 408 for the blind samples.

### Added dose&heating method

**Figure 5:**
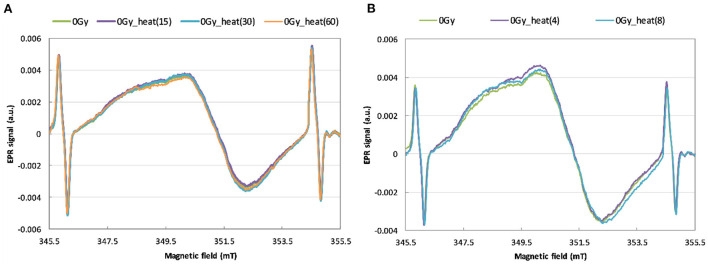
EPR spectra of unirradiated WG samples before and after annealing for 15, 30 and 60 min at 200 °C **(A)** and for 4 and 8 min at 250 °C **(B)**.

In order to use the heating method for reconstruction of an unknown dose, it is necessary to check whether the heating of a non-irradiated sample affects the intensity and shape of its EPR signal. Figures 5A,B show the effects of annealing of the unirradiated WG samples on their EPR spectra.

**Figure 6:**
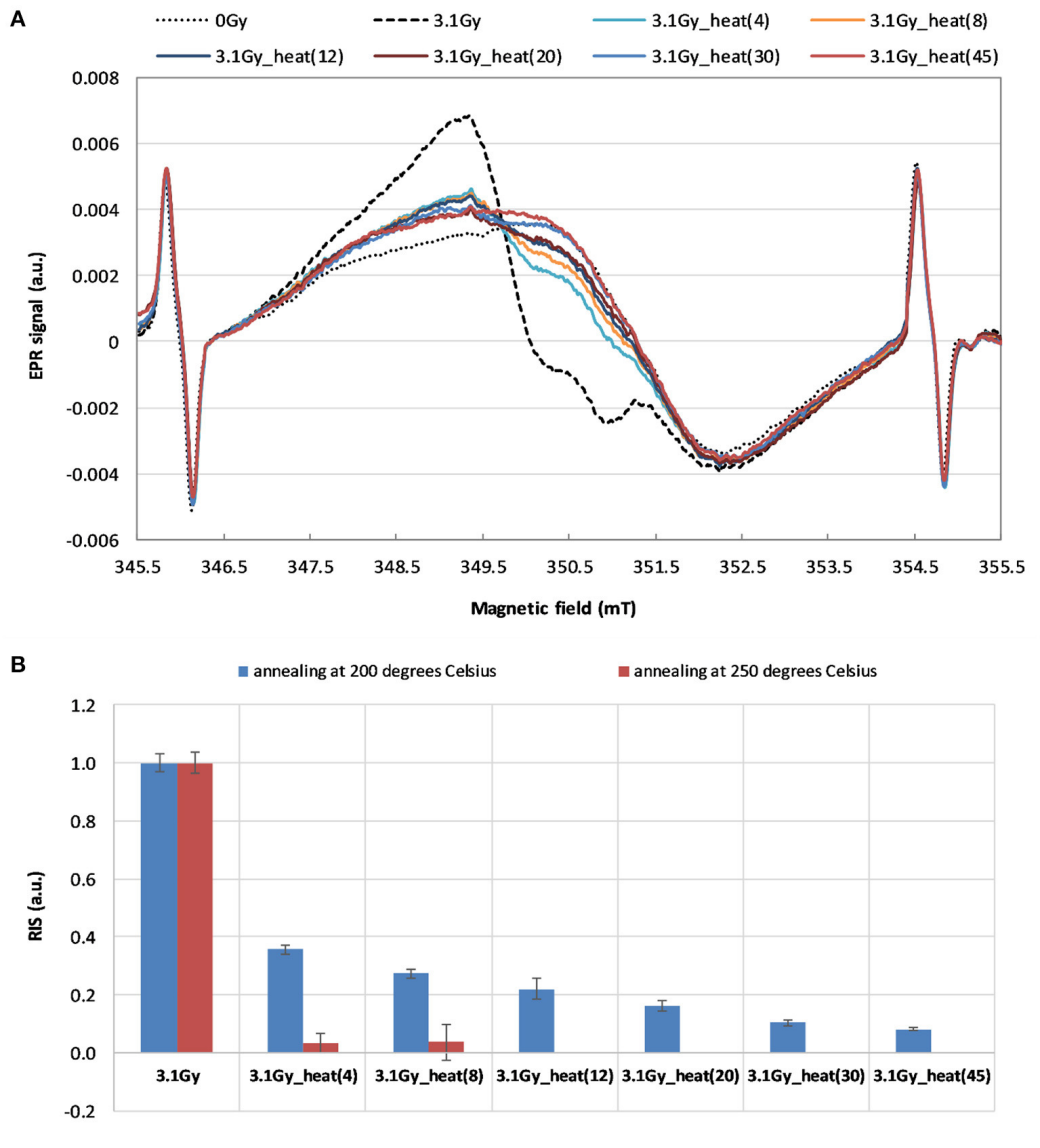
**(A)** The effect of time of annealing at 200 °C on the EPR spectra of the irradiated sample (3.1 Gy 232 in glass). **(B)** The dependence of the RIS component on duration of the annealing at 200 °C (for 4, 8, 12, 233 20, 30 and 45 min) and at 250 °C (for 4 and 8 min). The spectra in **(A)** and the data in **(B)** were marked according to headings of the respective columns in Table 2.

Figure 6 shows the effect of annealing at 200 °C and at 250 °C on spectra of the WG samples irradiated by 6 MVp photons with dose 3.1 *Gy* in glass. The changes in the EPR signal upon annealing up to 45 min is shown in Figure 6A. The corresponding decrease in the RIS component in time of the heating is shown in Figure 6B.

The EPR signal from the irradiated and annealed sample (spectra shown in Figure 6A) was used instead of the original BG (i.e., from unirradiated glass) in the numerical decomposition to reconstruct the dose by the calibration method (CM) and added dose method (ADM) for the three blind samples. The results are presented in Table 2 (columns 3–8). The real delivered doses are given in the second column (in terms of kerma in air).

**Table 2:** Comparison of the doses (in terms of kerma in air) reconstructed for 3 blind samples using a model BG signal recovered by heating of irradiated (3.1 Gy) sample for 4–45 min at 200 °C and for 4 and 8 min at 250 °C **(a)** by calibration method and **(b)** by added dose method.

**(a) Calibration method**							
**Annealing at 200** °**C**							
**Samples**	*K*_*air*_ **[Gy]**						
	**Real dose**	**Reconstructed dose using a model BG signal recovered by heating time (min)**					
		**heat (4)**	**heat (8)**	**heat (12)**	**heat (20)**	**heat (30)**	**heat (45)**
Blind 1	0	−1.7	−1.3	−1.1	−0.8	−0.6	−0.5
Blind 2	1.2	−0.4	0	0.2	0.5	0.7	0.8
Blind 3	3.5	1.8	2.1	2.4	2.6	2.8	2.9
**Annealing at 250** °**C**							
Blind 1	0	−0.3	−0.4				
Blind 2	1.2	1.0	0.9				
Blind 3	3.5	3.1	3.1				
**(b) Added dose method**							
**Annealing at 200** °**C**							
**Samples**	*K*_*air*_ **[Gy]**						
	**Real dose**	**Reconstructed dose using a model BG signal recovered by heating time (min)**					
		**heat (4)**	**heat (8)**	**heat (12)**	**heat (20)**	**heat (30)**	**heat (45)**
Blind 1	0	−2.81	−2.03	−1.54	−0.87	−0.40	−0.12
Blind 2	1.2	−1.45	−0.69	−0.23	0.34	0.71	0.94
Blind 3	3.5	1.58	2.27	2.68	3.23	3.57	3.76
**Annealing at 250** °**C**							
Blind 1	0	0.43	0.40				
Blind 2	1.2	1.35	1.32				
Blind 3	3.5	4.13	4.07				

The gray shadow indicates empty fields in the tables (with no data).

The dose reconstruction procedure by ADHM method, with recovery of the BG signal by annealing, was performed for one blind sample (Blind 3). It was first re-irradiated with a calibration dose *D*_*cal*_ = 5.17 *Gy*, measured and then heated for 10 min at 250 °C for determination of its BG signal. As a result, the dose of 3.19 Gy was reconstructed, as shown in the last column of Table 1.

## Discussion

As can be noticed in Figure 3, X-rays cause evident changes in the shape of EPR lines in the exposed WG samples. Analysis of the spectra presented in inset of Figure 3 proves that shape of the radiation-induced component is spectrally different than the BG signal. This indicates sensitivity of this type of glass to ionizing radiation and its potential applicability in retrospective dosimetry.

The dose dependence of the RISs for the WG samples measured up to 126 days after their irradiation for the purpose of calibration, are presented in Figure 4. The dose dependence is linear within the studied dose range (0–6 Gy). The rapid decay of slope of those calibration lines within the first 30 days after irradiation reflects a rapid decay of RIS. This is in accordance with observations of Juniewicz et al. (15), who also reported rapid decay of RIS within the first 10 days after irradiation. The greatest decay (about 30%) of the RIS was observed up to 50 days after irradiation. The signals measured 4 and 13 months later (on 126^th^ and 408^th^ day after irradiation) did not differ significantly from those measured on the 50^th^ day.

As can be noticed in Figure 5, no changes in shape and intensity of the spectra were observed after annealing up to 200 °C and 250 °C of the unirradiated watch glass samples. In contrary to our results obtained for WG, McKeever et al. (24) observed a rapid decay of the BG signal in Gorilla Glass samples at about 350–375 °C. Also Trompier et al. (10) observed an additional, heat-induced spectral component in spectra of glass substrates from mobile phones – in both irradiated and non-irradiated samples.

The data presented in Figures 6A,B shows that the 45 min of annealing at 200 °C reduces the RIS signal by more than 90%. Only 4 min of annealing the irradiated WG sample at 250 °C caused almost complete elimination of the RIS component (Figure 6B). Our results are consistent with the results described by Wu et al. (17) for watch glass, who showed that heating at 200 °C for one hour completely removed the dosimetric component. The results obtained in this study confirm a lack of influence of the annealing on the native BG signal of watch glass – neither on its shape nor intensity. The observed elimination of the RIS component in the irradiated samples' spectra by their annealing, together with resistance of the BG signal to annealing, are very advantageous features of the examined watch glass. This gives a unique possibility of reconstructing the dose absorbed in a real radiation accident, when the native background signal (BG) of the tested sample, which is necessary for accurate dose reconstruction, is not available. The ultimate verification of this approach in dose reconstruction is presented in Tables 1, 2 by comparison of the real and reconstructed doses.

From the data presented in Table 1 it can be concluded that the CM and ADM methods of dose reconstruction gave similar results. The reconstructed doses, with exception of the sample Blind 1 measured with ADM, differed at most by 0.34 Gy (about 10% in sample Blind 3, CM) from the real doses. The worst result was obtained for the sample Blind 1 using the ADM - the discrepancy between the real dose (0.0 Gy) and the reconstructed dose (0.63 Gy) resulted from very noisy spectra of this sample. The dose reconstructed by heating method in the Blind 3 sample was 3.19 Gy. An almost identical result was obtained with the calibration method (3.16 Gy).

Table 2 shows the doses reconstructed for the three blind samples using the calibration and the added dose procedures while EPR spectra measured in the annealed sample were used as the model BG signal instead of the original background from an unirradiated sample. As it could be expected, with increase in time of annealing the reconstructed doses were approaching the real ones. Annealing for 4 min at 250 degrees or for 20 min at 200 degrees allowed for reliable reconstruction of the dose in the sample Blind 3 (with 9% deviation from the real dose). It should be emphasized, that only the ADHM method can be used in a real scenario, when one has only one glass sample to measure, the one irradiated during the accident, and its specific BG spectrum is unknown.

Summarizing the presented results, it can be concluded that, the results obtained with the three tested methods show similar accuracy of about 0.3 Gy, sufficient for a reliable triage of people exposed in radiation accidents. Background signal of the examined watch glass was resistant to temperatures up to 250 °C, which gives the possibility to use the heating method to recover the background signal from irradiated samples in a real scenario. The ADHM is the only one, which allows to reconstruct absorbed dose if only one glass sample, the one irradiated during the accident, is available. Applicability of this method for other types of glasses requires verification of the background stability at high temperature. Moreover, as it results from previous reports (24, 25), an important factor disturbing a reliable dose reconstruction in glasses may be the effect of UV light. Therefore, additional research is necessary to assess applicability of the annealing in EPR dosimetry in glasses exposed to UV light.

## Data availability statement

The raw data supporting the conclusions of this article will be made available by the authors, without undue reservation.

## Author contributions

AM, MJ, and BC: EPR measurements, work concept, data analysis, and writing the manuscript. AP-D: reirradiation of samples and dosimetry. JK: Energy Dispersive Spectroscopy (EDS) measurement of elemental composition of watch glass samples. All authors contributed to the article and approved the submitted version.

## Funding

This work was supported by Medical University of Gdańsk, Internal Grant ST-70.

## Conflict of interest

The authors declare that the research was conducted in the absence of any commercial or financial relationships that could be construed as a potential conflict of interest.

## Publisher's note

All claims expressed in this article are solely those of the authors and do not necessarily represent those of their affiliated organizations, or those of the publisher, the editors and the reviewers. Any product that may be evaluated in this article, or claim that may be made by its manufacturer, is not guaranteed or endorsed by the publisher.

## Footnotes

^1^Abend Michael. Bundeswehr Institute of Radiobiology, Munich. Information distributed to participants of the RENEB 2021 Interlaboratory Comparison project; 2021.
